# The Effect of Ultra-Processed Food Consumption and Physical Activity on Fatty Acid Profiles in Young Muslims in Melilla

**DOI:** 10.3390/healthcare14101415

**Published:** 2026-05-21

**Authors:** Miriam Mohatar-Barba, María López-Olivares, Emilio González-Jiménez, Ana Rodríguez-Rodríguez, Mario Román-Cabezas, Javier S. Perona, Carmen Enrique-Mirón

**Affiliations:** 1 Department of Nursing, Faculty of Health Sciences, Melilla Campus, University of Granada, 52005 Melilla, Spain; miriamb@ugr.es; 2 Instituto de Investigación Biosanitaria (ibs. GRANADA), 18014 Granada, Spain; 3 Unit of Excellence of the University Campus of Melilla, University of Granada, 52005 Melilla, Spain; cenrique@ugr.es; 4 Department of Nutrition and Food Science, Faculty of Health Sciences, Melilla Campus, University of Granada, 52005 Melilla, Spain; mlopezolivares@ugr.es; 5 Department of Nursing, Faculty of Health Sciences, University of Granada, 18016 Granada, Spain; 6 Department of Clinical Biochemistry, Hospital Universitario Virgen del Rocío, 41013 Seville, Spain; ana.rodriguez.r.sspa@juntadeandalucia.es (A.R.-R.); mario.roman.sspa@juntadeandalucia.es (M.R.-C.); 7 Department of Food and Health, Institute of Fat-CSIC, Pablo de Olavide University Campus, Building 46, 41013 Seville, Spain; perona@ig.csic.es; 8 HUM-613 Research Group, Department of Inorganic Chemistry, Faculty of Health Sciences, Melilla Campus, University of Granada, C/Santander s/n, 52005 Melilla, Spain

**Keywords:** ultra-processed foods, adolescents, fatty acids, lipoproteins VLDL, erythrocyte membrane, physical activity

## Abstract

Background: The consumption of ultra-processed foods among adolescents is high due to their widespread availability and accessibility and has been linked to an increased cardiometabolic risk. In the Autonomous City of Melilla, it has been observed that Muslim adolescents consume more of these foods than their Christian peers, which warrants an exploratory analysis of their potential association with fatty acid biomarkers. Methods: A cross-sectional pilot study was conducted among 31 Muslim adolescents aged 15 to 17 years. The NOVA classification was used to identify the ultra-processed foods consumed, and the frequency of consumption, adherence to the Mediterranean diet, and level of physical activity were assessed. Fatty acid composition was determined in serum, VLDL, and erythrocytes. Results: Ultra-processed foods accounted for 49.1% of total daily energy intake, and 71% of the participants showed very low adherence to the Mediterranean diet. Saturated fatty acids predominated in VLDL, n-6 polyunsaturated fatty acids reached their highest levels in serum, and n-3 polyunsaturated fatty acids in erythrocytes. Furthermore, higher consumption of ultra-processed foods was positively correlated with saturated fatty acids in erythrocytes and with n-6 polyunsaturated fatty acids in VLDL and negatively correlated with n-3 polyunsaturated fatty acids in erythrocytes and VLDL. Conclusions: These findings suggest that more frequent consumption of these foods is associated with a less favorable fatty acid profile, which underscores the need for nutritional monitoring and preventive strategies tailored to the sociocultural context.

## 1. Introduction

Adolescence is a critical period for the establishment of eating habits and lifestyles, with implications that can extend into adulthood. In recent years, the Spanish youth population has shown a decline in adherence to the Mediterranean diet, with a lower presence of traditional foods and a higher consumption of pre-cooked foods of poorer nutritional quality. This shift in dietary patterns affects not only energy balance but also the type of fat incorporated into the daily diet [1,2].

In this context, the consumption of ultra-processed foods (UPFs) has increased significantly. Recent evidence from Spanish pediatric and adolescent populations has shown a progressive shift away from traditional Mediterranean dietary patterns, with lower adherence to the Mediterranean diet being associated with less favorable lifestyle behaviors and higher ultra-processed food intake [3]. In the Autonomous City of Melilla, a Spanish city located in North Africa where some of the world’s most influential religions coexist, high consumption of UPFs has been observed among adolescents, with differences associated with religion: Muslim schoolchildren have higher daily consumption of UPFs compared to their Christian peers [4,5,6]. This reality makes Melilla’s Muslim adolescent population a group of particular interest for research into whether the consumption of UPFs may be associated with metabolic changes.

From a nutritional standpoint, UPFs are characterized by their low content of fiber, protein, and micronutrients, and by being high in calories and rich in saturated and trans fats, added sugars, and sodium. In addition, they often contain flavorings, colorings, emulsifiers, and other additives that are particularly appealing to teenagers [7,8]. The available evidence in children and adolescents links higher consumption of these foods to poorer diet quality and an increased risk of excess body fat and cardiometabolic disorders, although the mechanisms remain the subject of ongoing research and may vary depending on the population studied and the pattern of consumption [9].

Furthermore, the body relies on the diet for its supply of essential fatty acids, particularly linoleic acid (n-6) and alpha-linolenic acid (n-3), due to their role in key structural and regulatory functions [10,11]. Specifically, linoleic acid and alpha-linolenic acid are considered essential fatty acids are considered essential because the human body cannot synthesize them and must obtain them through diet. Furthermore, these compounds act as precursors to other bioactive polyunsaturated fatty acids involved in development, cellular function, and metabolic regulation [12]. From this perspective, a balanced diet should not only meet energy needs but also ensure a healthy and adequate fat intake to maintain metabolic homeostasis.

However, high consumption of UPFs may be associated with a less favorable fatty acid profile by reducing the intake of polyunsaturated fatty acids of nutritional interest found in foods such as fish and nuts. Although the usefulness of the n-6/n-3 ratio remains a subject of debate, recent evidence suggests that it is more informative to assess adequate intake of long-chain omega-3s and the overall quality of dietary fat [13]. Furthermore, in adolescents with high consumption of UPFs, this interpretation may be particularly useful for understanding the potential link between dietary exposure and fatty acid biomarker profiles [14,15,16].

To analyze the potential relationship between the consumption of ultra-processed foods (UPFs) and differences in fatty acid biomarkers, it is useful to combine dietary information with biomarkers. Plasma or serum fractions provide a better indication of recent intake, while the erythrocyte membrane more reliably reflects the lipid profile over the medium term. Furthermore, the presence of eicosapentaenoic acid (EPA) and docosahexaenoic acid (DHA) in erythrocytes has frequently been used as an indicator of omega-3 intake [17,18,19]. Likewise, very-low-density lipoproteins (VLDL) are of great interest, as they transport triglycerides produced by the liver and play a role in the distribution of fatty acids to peripheral tissues [20].

Finally, physical activity among children and adolescents is considered a key component of the approach to cardiometabolic risk, as it may act as a modulator by influencing both fatty acid oxidation and the metabolism of triglyceride-rich lipoproteins through mechanisms that include increased lipoprotein lipase activity and improved insulin sensitivity [21,22]. However, few studies have jointly analyzed the consumption of UPFs, physical activity levels, and fatty acid profiles in adolescents; there are no data available for the Muslim adolescent population in Melilla, making this the first study conducted in a multicultural setting.

Previous research in this population has mainly described differences in UPF consumption according to sociodemographic and religious factors. However, to our knowledge, no previous study has examined whether this dietary exposure is reflected in fatty acid biomarkers measured in different biological compartments, such as serum, VLDL, and erythrocyte membranes. Therefore, the added value of the present study lies in combining dietary information with lipid biomarkers in a specific multicultural adolescent population that has been scarcely studied from a biochemical perspective.

Therefore, the objective of this pilot study was to analyze how the frequency of UPF consumption and physical activity levels are associated with the fatty acid composition of serum, VLDL, and erythrocyte membranes in Muslim adolescents in Melilla.

## 2. Materials and Methods

### 2.1. Study Design and Subjects

A pilot, observational, cross-sectional study was conducted on a purposive sample of 31 Muslim adolescents attending school in the Autonomous City of Melilla. Given the pilot and exploratory nature of the study, the sample was not intended to provide population-level estimates or to support broadly generalized conclusions. Rather, it was designed to provide a preliminary characterization of fatty acid biomarkers in a specific adolescent subgroup previously identified as having high exposure to UPFs. The mean age of the participants was 15.67 ± 0.62 years. The study was designed with a descriptive and exploratory approach, with the aim of characterizing the fatty acid profile in different biological compartments and analyzing its relationship with the consumption of ultra-processed foods (UPFs) and the level of physical activity.

### 2.2. Data Collection

**Figure 1:**
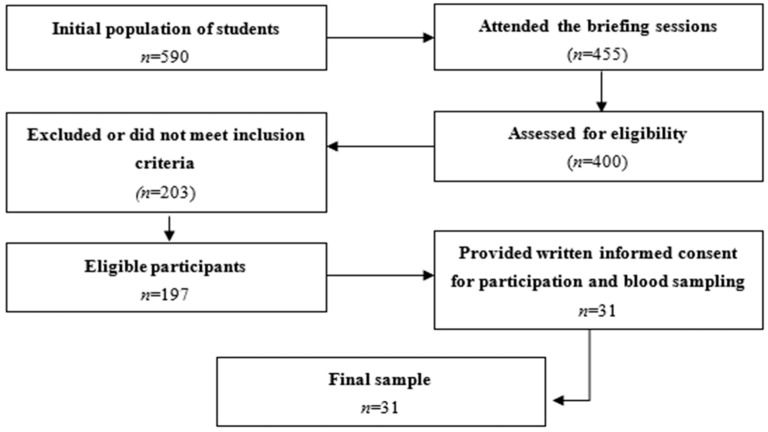
Flow diagram of the recruitment process.

The study was open to Muslim adolescents aged 15 to 17 who were enrolled in school in Melilla. Prior to their inclusion, informational sessions were held with parents or legal guardians to explain the study’s objectives, the planned tests, the questionnaires to be administered, and the blood collection procedure. Only those adolescents whose legal guardians provided written authorization for both study participation and blood sample collection were included. Adolescents without informed consent were excluded, as were those with any condition that would prevent the measurements outlined in the protocol from being performed. Figure 1 summarizes the recruitment process, including the number of adolescents who met these criteria for blood sampling.

In May 2024, informational sessions were held with parents or legal guardians at various schools, during which the study’s characteristics and the planned assessments were explained. During these sessions, anthropometric data, body composition measurements, blood pressure readings, dietary information, and physical activity data were collected from the participants. Subsequently, between June and July 2024, blood samples were collected after an overnight fast, and the samples were sent to the Instituto de la Grasa (CSIC, Seville) for analysis. Biochemical processing was carried out in August 2024.

The study was conducted in accordance with the principles established in the Declaration of Helsinki [23] and has been approved by the Research Ethics Committee of the University of Granada (Code 2752/CEIH/2022), approved on 8 November 2022. Specific information regarding this approval is provided in the section on ethical considerations.

### 2.3. Blood Pressure

Blood pressure was measured in the morning, in a quiet room, using a previously calibrated sphygmomanometer and a Littmann^®^ stethoscope (3M Company, St. Paul, MN, USA), in accordance with the recommendations of the American Heart Association [24]. Participants remained seated for at least 5 min before measurement, with their back supported, feet flat on the floor, legs uncrossed, and the arm supported at heart level. Measurements were taken in the seated position, and participants were instructed not to talk during the procedure.

### 2.4. Dietary Intake

Dietary assessment was conducted using a food frequency questionnaire (FFQ), the KIDMED Mediterranean Diet Adherence Questionnaire, and a 72-h dietary recall.

Food frequency was assessed using a 44-item food frequency questionnaire, which allowed for the recording of the usual frequency of consumption for each food (daily, weekly, or monthly) [25]. In addition, a 72-h dietary recall was used, covering three consecutive days (Thursday, Friday, and Saturday), in order to record food intake throughout the week and identify any differences between weekdays and weekends [26]. Both instruments have been previously validated for use by adolescents.

Dietary information was collected through in-person interviews conducted by trained staff, during which the foods, beverages, and supplements consumed over the three-day assessment period were recorded. To facilitate the estimation of the amounts consumed, standardized household measures and reference images were used.

Adherence to the Mediterranean diet was assessed using the KIDMED questionnaire, which was validated in Spain in its original version and subsequently updated [27,28]. This instrument consists of 16 dichotomous (yes/no) questions that assess dietary patterns related to the Mediterranean diet. Eight items reflect favorable habits, such as the consumption of fruits, vegetables, fish, legumes, or dairy products, and are scored as +1, while the remaining eight assess less healthy behaviors, such as the consumption of industrial pastries, fast food, or skipping breakfast, subtracting −1 point when the answer is affirmative. The total score ranges from −4 to 12 points, allowing adherence to the Mediterranean diet to be classified as low (≤3 points), moderate (4–7 points), and high (≥8 points).

In addition, total energy intake and macro- and micronutrient content were estimated using the dietary calculator from the Institute of Endocrinology and Nutrition of Valladolid (IENVA) (https://calcdieta.ienva.org/tu_menu.php accessed on 1 February 2024). Based on the data obtained from the FFQ and the 72-h dietary recall, foods and beverages classified as NOVA group 4 were identified, and their contribution to total daily energy intake was calculated and expressed as the percentage of daily energy derived from UPFs. Dietary fatty acid intake was also estimated from the 72-h dietary recall using the IENVA dietary calculator and expressed as grams per day. Therefore, the dietary fatty acid values reported below represent estimated daily intake rather than the relative percentage of total fatty acids.

Finally, the foods and beverages recorded in the dietary questionnaires were classified according to the NOVA system [29], which categorizes foods based on their degree and purpose of industrial processing into: unprocessed or minimally processed foods, processed culinary ingredients, processed foods, and ultra-processed foods. This classification made it possible to identify the UPFs consumed and estimate the energy and key nutrients provided by this food group for each participant.

### 2.5. Anthropometric Measurements

Anthropometric measurements included height, weight, waist circumference, and hip circumference, and were performed in accordance with the standards of the International Society for the Advancement of Kinanthropometry (ISAK) [30]. Body composition was determined using electrical bioimpedance analysis with a TANITA^®^ SC-330 (Tanita Corporation, Tokyo, Japan) device.

### 2.6. Physical Activity

Physical activity was assessed using the short version of the International Physical Activity Questionnaire (IPAQ), which has been validated for individuals aged 15 to 69 [31]. Total physical activity was expressed in MET-min/week, in accordance with the questionnaire’s scoring protocol, and participants were classified into low, moderate, or high levels of physical activity based on established cutoff points.

Although the dietary and physical activity instruments used in this study have been validated or previously used in Spanish adolescent populations, they have not been specifically validated in Spanish Muslim adolescents. Therefore, all questionnaires were administered by trained researchers using standardized procedures, including face-to-face interviews, standardized household measures, and reference images to improve the accuracy of dietary reporting.

### 2.7. Biochemical Analysis

Venous blood samples were collected by qualified personnel between 8:00 and 9:00 a.m. after a standardized 12-h overnight fast. Participants and their legal guardians received written and verbal instructions before sample collection. During the fasting period, participants were allowed to drink water only and were instructed to avoid tea, coffee, sugar-sweetened beverages, energy drinks, and any caloric intake. They were also asked to avoid vigorous physical activity during the previous 24 h. However, maintenance of the usual diet during the three weeks prior to blood sampling was not objectively verified, and this has been acknowledged as a limitation.

**Figure 2:**
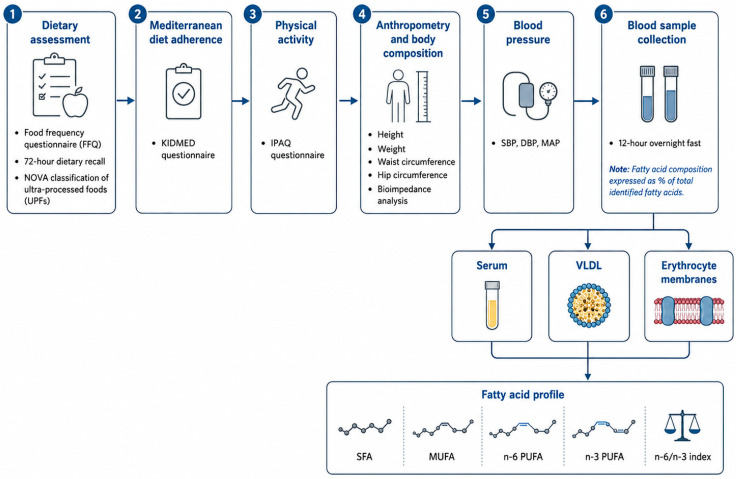
Overview of study assessments and biochemical parameters.

A total of 10 mL of venous blood was drawn from the antecubital vein using single-use vacuum blood collection tubes. Blood samples were processed to obtain serum, VLDL fractions, and erythrocyte membranes. Fatty acid composition was determined in these biological compartments and expressed as the percentage of total identified fatty acids. The main fatty acid groups analyzed were saturated fatty acids, monounsaturated fatty acids, n-6 polyunsaturated fatty acids, n-3 polyunsaturated fatty acids, and the n-6/n-3 index. A schematic overview of the study assessments, biological samples, and biochemical parameters analyzed is presented in Figure 2.

After collection, blood samples were processed on the same day to obtain serum, VLDL fractions, and erythrocyte membranes. Samples were aliquoted to minimize repeated freeze–thaw cycles and stored at −20 °C until transfer to the Instituto de la Grasa (CSIC, Seville) for fatty acid analysis. Information on the use of additional antioxidants or preservatives was not available.

Fatty acid composition was analyzed by the specialized laboratory of the Instituto de la Grasa (CSIC, Seville) following internal laboratory protocols. Individual fatty acids were identified and quantified in serum, VLDL fractions, and erythrocyte membranes, and results were expressed as the percentage of total identified fatty acids in each biological fraction. Quality control procedures were conducted according to the laboratory’s internal analytical standards. However, detailed reproducibility coefficients were not available for inclusion in the manuscript, and this has been acknowledged as a limitation.

### 2.8. Statistical Analysis

Statistical analysis was performed using IBM SPSS Statistics, version 28.0. Quantitative variables were expressed as the mean ± standard deviation (SD) when normally distributed, and as the median [interquartile range] when non-normally distributed or when non-parametric analyses were applied. Qualitative variables were presented as absolute frequencies and percentages.

The choice of statistical tests was based on the exploratory nature of the pilot study and on the type and distributional characteristics of the variables analyzed. The FFQ for UPFs and beverages of interest was summarized using contingency tables, and consumption was categorized into three groups: never/monthly, weekly, and daily. The fatty acid composition in serum, VLDL, and erythrocyte membrane phospholipids was expressed as a percentage of the total fatty acids identified in each biological fraction.

Comparisons between paired biological fractions, specifically serum and erythrocyte membrane phospholipids, were performed using the Wilcoxon signed-rank test. The relationship between UPF consumption, physical activity level, and fatty acids in VLDL, erythrocytes, and serum was explored using Spearman’s rank correlation coefficient. No multivariable adjusted models were fitted because of the limited sample size and the exploratory nature of the study, as the inclusion of several covariates would have resulted in unstable estimates and a high risk of overfitting. Therefore, the reported associations should be interpreted as unadjusted exploration associations. The strength of the correlations was interpreted according to Schober et al. [32], considering correlation coefficients of 0.00–0.10 as negligible, 0.10–0.39 as weak, 0.40–0.69 as moderate, 0.70–0.89 as strong, and 0.90–1.00 as very strong.

In addition, PUFA levels were compared according to physical activity level categories (low, moderate, and high) using the Kruskal–Wallis test. Effect size for Kruskal–Wallis analyses were estimated using epsilon-squared (ε^2^). Pairwise post hoc comparisons were planned to use the Dunn–Bonferroni method when statistically significant differences were detected.

Given the exploratory nature of this pilot study, no formal adjustment for multiple comparisons was applied. Therefore, correlation analyses were interpreted cautiously as exploratory findings rather than confirmatory evidence. In all analyses, a *p*-value < 0.05 was considered statistically significant.

## 3. Results

### 3.1. General Characteristics of the Sample

Table 1 shows the anthropometric characteristics, nutritional status, blood pressure, and physical activity levels of the participants in the pilot study. The sample consisted of 31 Muslim adolescents with a mean age of 15.67 ± 0.62 years. Regarding nutritional status, 54.8% were of normal weight, while 25.8% had excess weight, including 16.1% with overweight and 9.7% with obesity. Most participants did not present cardiometabolic risk according to the waist-to-height ratio (93.5%) and had normal blood pressure levels (83.9%). Regarding physical activity, nearly half of the sample was at a high level (48.4%). Furthermore, the mean KIDMED index score was 2.87 ± 2.39, indicating low adherence to the Mediterranean diet.

### 3.2. Frequency of Ultra-Processed Food Consumption and Dietary Fatty Acid Composition

Table 2 shows the frequency of consumption of UPFs in the study sample. High daily consumption was observed for several products, particularly industrial juices (48.4%), sweets and candy (45.2%), industrial sauces (45.2%), bagged potato chips (41.9%), salty snacks (41.9%), and chocolate (41.9%). Other products, such as industrial pastries and packaged milkshakes, also showed a significant daily consumption frequency (32.3%).

Table 3 shows the frequency of consumption of sugary, isotonic, energy, and caffeinated beverages. Among these, sugary soft drinks stood out, consumed daily by 45.2% of participants, followed by energy drinks (38.7%). Daily consumption of sports drinks was lower (16.1%), as was that of diet soft drinks (12.9%) and coffee (9.7%).

Overall, the average energy intake from the diet was 2382.11 ± 449.11 kcal/day, and energy from NOVA group 4 ultra-processed foods accounted for 49.1% of total daily energy intake, indicating that nearly half of the energy consumed by the participants came from UPFs. Furthermore, adherence to the Mediterranean diet was low in the majority of the sample, with 71% of adolescents classified in the very low adherence category, 22.6% in the intermediate adherence category, and only 6.5% in the optimal adherence category.

On the other hand, Table 4 shows the estimated dietary fatty acid intake expressed in g/day. Among saturated fatty acids, palmitic acid was the predominant one (17.26 ± 6.32 g/day), followed by stearic acid (5.83 ± 1.58 g/day). Among monounsaturated fatty acids, oleic acid showed the highest intake (44.06 ± 13.30 g/day). Among polyunsaturated fatty acids, linoleic acid was the main representative of the n-6 series (11.74 ± 3.91 g/day), while the estimated intakes of long-chain n-3 fatty acids, such as EPA and DHA, were low (0.10 ± 0.12 and 0.11 ± 0.17 g/day, respectively).

### 3.3. Composition of Circulating and Erythrocyte Membrane Fatty Acids

Table 5 shows the fatty acid composition of VLDL, erythrocyte membrane phospholipids, and serum in the 31 adolescents included in the pilot study. Overall, differences were observed in the distribution of the major fatty acid groups depending on the biological fraction analyzed.

Saturated fatty acids (SFAs) constituted the largest group in VLDL, accounting for an average of 44.95 ± 11.06% of total fatty acids. Among these, palmitic acid was the predominant SFA in all three fractions, particularly in serum (28.08 ± 2.60%) and VLDL (25.62 ± 9.84%), while stearic acid showed higher values in VLDL (18.33 ± 4.80%) than in erythrocytes and serum.

Regarding monounsaturated fatty acids (MUFA), oleic acid was the main representative of this group in all three fractions.

n-6 polyunsaturated fatty acids (n-6 PUFAs) reached their highest mean values in serum (37.04 ± 3.33%), followed by erythrocytes (32.24 ± 6.64%) and VLDL (27.14 ± 8.01%). Within this family, linoleic acid was clearly more abundant in serum (25.87 ± 3.29%) than in VLDL and erythrocytes, while arachidonic acid had its highest proportion in erythrocytes (13.30 ± 3.15%), higher than in serum and VLDL.

Meanwhile, n-3 polyunsaturated fatty acids (n-3 PUFAs) showed a different distribution, with the highest values in erythrocytes (7.96 ± 1.95%), compared to VLDL and serum. In this erythrocyte fraction, EPA (3.14 ± 0.93%) and DHA (3.13 ± 1.03%) were particularly prominent. Consequently, the n-6/n-3 ratio was lower in erythrocytes (4.14 ± 0.69) than in VLDL and serum.

Taken together, these results reflect a differential distribution of fatty acids among the different fractions analyzed, with a relative predominance of SFA in VLDL, a higher proportion of n-6 PUFA in serum, and a higher presence of n-3 PUFA in erythrocyte membrane phospholipids.

On the other hand, Table 6 shows the comparison between the percentages of fatty acids in serum and in erythrocyte membrane phospholipids. Significant differences were observed in several compounds, with higher values in serum for palmitic acid and linoleic acid, and higher values in erythrocytes for myristic acid, docosanoic acid, arachidonic acid, 18:3 (n-3), and DHA. No significant differences were detected for stearic acid, 20:2 (n-6), DHGLA, 22:4 (n-3), or DPA. Taken together, these findings indicate that serum and erythrocytes provide complementary information on the lipid profile, as the former appears to better reflect recent dietary exposure, while membrane phospholipids offer a more stable estimate with a greater metabolic component.

### 3.4. Association Between the Frequency of UPF Consumption and Physical Activity with Circulating and Membrane Fatty Acids

Table 7 shows the correlations between the frequency of ultra-processed food consumption, physical activity levels, and the sum of fatty acids in VLDL, erythrocytes, and serum. The frequency of UPF consumption was associated with various groups of fatty acids in the different biological fractions. Specifically, positive correlations were observed with saturated fatty acids in erythrocytes and with n-6 polyunsaturated fatty acids in VLDL, as well as negative correlations with monounsaturated fatty acids in serum and with n-3 polyunsaturated fatty acids in VLDL and erythrocytes. In contrast, no statistically significant associations were identified between the level of physical activity and the fatty acids analyzed.

In addition, PUFA levels were compared according to physical activity level categories (low, moderate, and high). As shown in Table 8, no statistically significant differences were observed in ∑n-6 PUFA, ∑n-3 PUFA, or the n-6/n-3 index in VLDL, erythrocyte membranes, or serum across physical activity groups. Therefore, no post hoc pairwise comparisons were made.

## 4. Discussion

The aim of this pilot study was to describe the fatty acid profile in different biological compartments and to explore its relationship with the consumption of ultra-processed foods (UPFs) and the level of physical activity in a sample of Muslim adolescents from Melilla. This approach is particularly relevant because it focuses on a subgroup previously identified as having high exposure to UPFs and allows for a descriptive and exploratory examination of possible differences in fatty acid biomarkers associated with dietary patterns. In this regard, our results show, on the one hand, a high frequency of consumption of UPFs and sugar-sweetened or energy drinks and, on the other, a differential fatty acid profile depending on the biological fraction analyzed, with consistent associations between the frequency of UPFs consumption and various circulating and membrane fatty acids.

First, the frequency of consumption observed for various UPFs reinforces the notion of a dietary pattern that deviates from healthy dietary recommendations. In our sample, daily consumption of commercial juices, sweets and snacks, commercial sauces, salty snacks, and bagged potato chips stood out, along with a high frequency of sugary soft drinks and energy drinks. Furthermore, nearly half of daily energy intake came from UPFs, and adherence to the Mediterranean diet was low among most participants. This pattern is consistent with recent literature, which identifies UPFs as a common component of children’s and adolescents’ diets and links them to poorer overall diet quality and increased cardiometabolic risk [9,33,34,35].

From a nutritional standpoint, the fatty acid composition of the diet showed a predominance of palmitic acid among saturated fatty acids, oleic acid among monounsaturated fatty acids, and linoleic acid among n-6 polyunsaturated fatty acids, while EPA and DHA intakes were low. This distribution suggests a diet with a significant presence of saturated and n-6 fats, but with a low intake of long-chain n-3s, which is consistent with low consumption of fresh foods rich in omega-3s, such as oily fish or nuts, and with a greater reliance on processed foods. Although the n-6/n-3 ratio should not be interpreted in isolation, current evidence suggests that low dietary exposure to EPA and DHA during adolescence may have metabolic and cardiovascular implications [11,12].

The fatty acid composition observed in the different biological fractions showed a differential pattern. VLDLs had a higher relative proportion of saturated fatty acids, serum had higher levels of n-6 polyunsaturated fatty acids, particularly linoleic acid, and erythrocytes had the highest proportions of n-3 polyunsaturated fatty acids, along with notable levels of arachidonic acid. This finding is consistent with a dietary pattern characterized by a high presence of saturated fats and n-6 fatty acids, as well as a lower intake of long-chain n-3 fatty acids. Furthermore, the higher presence of arachidonic acid in erythrocytes and the reduced levels of EPA and DHA in certain fractions could indicate a less favorable lipid profile from an inflammatory and cardiometabolic standpoint. In this regard, the results reinforce the utility of considering separately the information provided by serum, VLDL, and erythrocyte membranes when studying the metabolic impact of diet in adolescents [36,37,38].

Furthermore, the comparison between serum and erythrocyte membrane phospholipids supports this interpretation. In our study, palmitic acid and linoleic acid were higher in serum, while arachidonic acid and DHA were higher in erythrocytes. This pattern is consistent with that described in a healthy pediatric population, where serum levels have been more closely linked to recent dietary intake, whereas erythrocyte membranes reflect a longer-term integration and a greater influence of endogenous metabolism [11,39,40]. Thus, the differences observed between the two fractions should not be interpreted as discrepancies, but rather as an expression of two distinct levels of biological information.

One of the relevant exploratory findings of this study was the association between the frequency of UPF consumption and fatty acid composition in the various fractions analyzed. Specifically, higher UPF consumption was positively correlated with saturated fatty acids in erythrocytes and with n-6 polyunsaturated fatty acids in VLDL, and negatively correlated with n-3 polyunsaturated fatty acids in erythrocytes and VLDL [41,42,43]. This pattern may be relevant because it suggests not only greater dietary exposure to lower-quality fats, but also a lower relative intake of long-chain n-3 fatty acids, which are typically associated with a healthier metabolic profile [37,38]. Furthermore, the composition observed in red blood cells provides a more stable reflection of the body’s lipid composition, rather than merely its most recent intake [39,40].

These associations may be compatible with a less favorable fatty acid profile among adolescents with higher UPF consumption. However, given the cross-sectional design, small sample size, and the number of exploratory correlations performed, these findings should be interpreted cautiously and should not be considered evidence of causality or definitive metabolic alteration. Rather, they should be viewed as preliminary hypothesis-generating results that require confirmation in larger longitudinal studies.

Regarding physical activity, our results did not show significant differences in PUFA levels according to low, moderate, or high physical activity categories. Likewise, physical activity showed no consistent associations with PUFA-related biomarkers in serum, VLDL, or erythrocyte membranes. In contrast, the frequency of UPF consumption showed more consistent associations with a less favorable fatty acid profile, including higher saturated fatty acids in erythrocytes, higher n-6 PUFAs in VLDL, and lower n-3 PUFAs in VLDL and erythrocyte membranes. These findings suggest that, in this pilot sample, dietary exposure to UPFs may be more closely related to fatty acid biomarkers than self-reported physical activity level. However, this interpretation should be made cautiously, as the absence of significant differences according to physical activity may be influenced by the limited sample size, the cross-sectional design, and the use of self-reported physical activity data. The lack of significant findings for physical activity may also reflect the limited variability and small number of participants in each activity category, as well as the fact that self-reported questionnaires may not capture intensity, duration, and patterns of activity with sufficient precision to detect subtle associations with fatty acid biomarkers. For this reason, future studies should consider objective physical activity measures, such as accelerometry.

From a public health perspective, these findings may help guide strategies aimed at improving eating behaviors among adolescents. In this sample, the high frequency of consumption of industrial juices, sweets, salty snacks, industrial sauces, sugar-sweetened beverages, and energy drinks suggests the need for interventions focused on reducing the availability and habitual intake of these products. At the same time, school- and family-based programs should promote feasible and culturally adapted alternatives, such as increasing the intake of fruits, vegetables, legumes, nuts, olive oil, and fish, within the framework of a Mediterranean dietary pattern. In multicultural settings such as Melilla, nutritional education should consider the sociocultural and religious context of adolescents and their families in order to improve adherence and long-term effectiveness.

In general terms, our findings support the utility of combining dietary indicators with biomarkers to study the metabolic impact of dietary patterns in adolescents. In populations with high consumption of UPFs, the analysis of diet and the composition of fatty acids in serum, VLDL, and erythrocytes may provide a clearer picture than dietary assessment alone. This strategy is particularly valuable during adolescence, a stage in which eating habits are established with the potential for long-term impact on cardiometabolic risk [44].

Finally, this study should be interpreted with several limitations in mind. First, this was a pilot, cross-sectional study based on a small purposive/convenience sample, which limits statistical power, causal inference, and the generalizability of the findings beyond this specific subgroup of Muslim adolescents in Melilla. Second, the small sample size reduces statistical power and increases the risk of type II error, particularly in subgroup analyses according to physical activity level. In addition, because adjusted multivariable analyses were not performed, potential confounding by sex, BMI, total energy intake, body fat, adherence to the Mediterranean diet, or physical activity cannot be excluded. Furthermore, no formal correction for multiple comparisons was applied, which may increase the risk of false-positive findings; therefore, statistically significant correlations should be interpreted with caution. Third, dietary intake and physical activity were assessed using self-reported instruments, which may be affected by recall bias and social desirability bias. Moreover, although these questionnaires have been validated or previously used in Spanish adolescent populations, they have not been specifically validated in Spanish Muslim adolescents, which may limit their cultural sensitivity and precision in this specific population. Fourth, although blood samples were collected after an overnight fast using a standardized protocol, some preanalytical factors, such as the maintenance of the usual diet in the weeks before blood sampling or the avoidance of major lifestyle changes, could not be fully verified. Finally, the study focused on a very specific group of adolescents in Melilla, which represents both a limitation in terms of external validity and strength, as this population has been scarcely studied in relation to ultra-processed food consumption and fatty acid biomarkers.

In addition, although samples were stored frozen until analysis, detailed information on storage duration and the use of antioxidants or preservatives was limited; therefore, potential oxidation-related preanalytical effects cannot be completely excluded. Moreover, detailed laboratory reproducibility indicators, such as intra- and inter-assay coefficients of variation, were not available, which may limit the assessment of analytical precision.

Future studies should aim to improve the robustness of the evidence by including larger and more representative samples, preferably selected through probabilistic sampling strategies. Longitudinal designs would also be useful to clarify the temporal relationship between ultra-processed food consumption, physical activity, and changes in fatty acid profiles. In addition, future research should incorporate objective measures of physical activity, such as accelerometry, repeated dietary assessments, and repeated blood sampling to better capture intra-individual variability. The use of culturally adapted and specifically validated questionnaires for Spanish Muslim adolescents would further strengthen the accuracy of dietary and lifestyle assessment in this population.

## 5. Conclusions

In conclusion, this pilot study suggests that a higher frequency of UPF consumption among Muslim adolescents in Melilla may be associated with a less favorable circulating and membrane fatty acid profile, characterized by a higher proportion of saturated and n-6 polyunsaturated fatty acids and a lower presence of n-3 polyunsaturated fatty acids. These preliminary findings reinforce the potential utility of combining dietary assessment with lipid biomarkers, such as the fatty acid composition of serum, VLDL, and erythrocyte membranes, to better understand diet-related metabolic profiles during adolescence. However, given the cross-sectional and exploratory nature of the study, these results should be interpreted as associations rather than evidence of causality or early lipid metabolism alterations. Larger longitudinal studies in multicultural adolescent populations are needed to confirm these findings and to clarify the temporal relationship between UPF consumption, physical activity, and fatty acid profiles.

## Figures and Tables

**Table 1:** Anthropometric characteristics, nutritional status, blood pressure, and physical activity levels of the pilot sample.

		All (*n* = 31)
Age (years)		15.67 ± 0.62
Height (cm)		167.87 ± 8.70
Weight (kg)		63.09 ± 15.63
BMI (kg/m^2^)		22.19 ± 4.69
**Nutritional status**		
	Underweight	6 (19.4)
	Normal weight	17 (54.8)
	Overweight	5 (16.1)
	Obese	3 (9.7)
Waist circumference (cm)		72.53 ± 9.96
Hip circumference (cm)		97.58 ± 10.35
WHI		0.74 ± 0.06
WHT		0.43 ± 0.05
**Cardiometabolic risk**		
	No risk (ICA < 0.5)	29 (93.5)
	At risk (ICA > 0.5)	2 (6.5)
Fat mass (kg)		12.93 ± 8.06
Body fat (%)		19.18 ± 9.71
Lean mass (kg)		50.53 ± 11.39
Muscle mass (kg)		47.60 ± 10.47
Heart rate		78.48 ± 9.85
SBP (mmHg)		115 ± 17.63
DBP (mmHg)		74.06 ± 13.90
MAP (mmHg)		94.83 ± 14.32
**BP level**		
	Normal	26 (83.9)
	Prehypertensive	3 (9.7)
	Hypertensive	2 (6.5)
**Physical activity level**		
	Low	6 (19.4)
	Moderate	10 (32.3)
	High	15 (48.4)
**KidMED Index**		2.87 ± 2.39

Note. Data are presented as the mean ± SD and frequency (percentages, %), depending on the variable. BMI: Body mass index; WHI: Waist-hip index; WHT: Waist-height index; SBP: Systolic blood pressure; DBP: Diastolic blood pressure; MAP: Mean arterial pressure.

**Table 2:** Frequency of consumption of ultra-processed foods.

	All (*n* = 31)		
Ultra-Processed Foods	Never/Monthly	Weekly Consumption	Daily Consumption
Industrial pastries (bollicaos, donuts, etc.)	6 (19.4)	15 (48.4)	10 (32.3)
Bagged potato chips	8 (25.8)	10 (32.3)	13 (41.9)
Salty snacks (Gusanitos, Cheetos)	3 (9.7)	15 (48.4)	13 (41.9)
Industrial hamburgers	11 (35.5)	18 (58.1)	2 (6.5)
Cold cuts (ham, turkey)	16 (51.6)	10 (32.3)	5 (16.1)
Sausages (salchichón, chorizo, etc.)	10 (32.3)	16 (51.6)	5 (16.1)
Smoked sausages and other smoked cured meats	16 (51.6)	10 (32.3)	5 (16.1)
Candy/sweets	5 (16.1)	12 (38.7)	14 (45.2)
Packaged milkshakes	6 (19.4)	15 (48.4)	10 (32.3)
Industrial juices	4 (12.9)	12 (38.7)	15 (48.4)
Chocolate	4 (12.9)	14 (45.2)	13 (41.9)
Industrial sauces (ketchup, mayonnaise)	3 (9.7)	14 (45.2)	14 (45.2)

**Table 3:** Frequency of consumption of sugar, sports, energy, and caffeinated beverages.

	All (*n* = 31)		
Beverages	Never/Monthly	Weekly Consumption	Daily Consumption
Sugary soft drinks (Coca-Cola, etc.)	7 (22.6)	10 (32.3)	14 (45.2)
Diet soft drinks	12 (38.7%)	15 (48.4)	4 (12.9%)
Sports drinks (Aquarius, Isostar, etc.)	12 (38.7)	14 (45.2)	5 (16.1)
Energy drinks (Red Bull, Monster, etc.)	10 (32.3)	9 (29.0)	12 (38.7)
Coffee	20 (64.5)	8 (25.8)	3 (9.7)

**Table 4:** Dietary fatty acid intake (g/day).

Fatty Acid		Mean ± SD
**SFAs**	12:0 (Lauric acid)	1.87 ± 3.20
	14:0 (Myristic acid)	2.25 ± 1.40
	16:0 (Palmitic acid)	17.26 ± 6.32
	18:0 (Stearic acid)	5.83 ± 1.58
**MUFA**	18:1 (n-9) (Oleic acid)	44.06 ± 13.30
**PUFA-n6**	18:2 (n-6) (Linoleic acid)	11.74 ± 3.91
**PUFA-n3**	18:3 (n-3)	0.65 ± 0.21
	20:5 (n-3) (EPA, Eicosapentaenoic acid)	0.10 ± 0.12
	22:6 (n-3) (DHA, Docosahexaenoic acid)	0.11 ± 0.17
**CIS**		47.34 ± 13.91
**TRANS**		1.41 ± 0.48

Note. Data are presented as the mean ± SD and expressed as g/day, calculated from the 72-h dietary recall. SFA: saturated fatty acids; MUFA: monounsaturated fatty acids; PUFA: polyunsaturated fatty acids.

**Table 5:** Fatty acid composition of very-low-density lipoproteins (VLDL), red blood cells, and serum in the participants.

		All (*n* = 31)		
	Fatty Acid	VLDL	Red Blood Cells	Serum
**SFAs**	12:0 (Lauric acid)	--	--	0.14 ± 0.38
	14:0 (Myristic acid)	0.34 ± 0.77	0.49 ± 0.35	0.34 ± 0.96
	16:0 (Palmitic acid)	25.62 ± 9.84	21.20 ± 4.60	28.08 ± 2.60
	18:0 (Stearic acid)	18.33 ± 4.80	11.99 ± 2.70	11.25 ± 4.72
	20:0 (Arachidic acid)	--	0.01 ± 0.04	--
	22:0 (Docosanoic acid)	--	0.60 ± 0.39	0.03 ± 0.09
	∑SFA (% of total fatty acids)	44.95 ± 11.06	34.34 ± 6.99	39.50 ± 4.17
**MUFA**	14:1 (n-5)	0.92 ± 0.97	0.24 ± 0.26	0.51 ± 0.37
	16:1 (n-7)	1.86 ± 1.67	0.28 ± 0.23	--
	16:1 (n-9)	--	1.20 ± 2.08	0.32 ± 0.81
	18:1 (n-9) (Oleic acid)	19.81 ± 8.55	16.96 ± 4.96	15.70 ± 2.49
	20:1 (n-9) (Eicosenoic acid)	--	0.09 ± 0.16	--
	∑MUFA (% of total fatty acids)	22.37 ± 8.59	18.61 ± 5.19	16.53 ± 3.04
**PUFA-n6**	18:2 (n-6) (Linoleic acid)	18.50 ± 6.40	15.47 ± 3.60	25.87 ± 3.29
	18:3 (n-6)	--	1.75 ± 1.13	--
	20:2 (n-6)	--	0.14 ± 0.19	0.16 ± 0.19
	20:3 (n-6) (DHGLA, dihomo-γ-linolenic acid)	1.90 ± 1.26	1.57 ± 0.67	1.96 ± 0.82
	20:4 (n-6) (AA, Arachidonic acid)	6.73 ± 2.59	13.30 ± 3.15	9.04 ± 2.03
	∑n-6 PUFAs (% of total fatty acids)	27.14 ± 8.01	32.24 ± 6.64	37.04 ± 3.33
**PUFA-n3**	18:3 (n-3)	2.30 ± 2.25	0.45 ± 0.68	0.02 ± 0.07
	20:5 (n-3) (EPA, eicosapentaenoic acid)	0.77 ± 0.94	3.14 ± 0.93	--
	22:3 (n-3)	--	--	0.88 ± 0.71
	22:4 (n-3)	0.43 ± 0.41	0.22 ± 0.50	0.22 ± 0.38
	22:5 (n-3) (DPA, docosapentaenoic acid)	0.23 ± 0.28	1.03 ± 0.55	0.96 ± 0.83
	22:6 (n-3) (DHA, Docosahexaenoic acid)	1.79 ± 1.06	3.13 ± 1.03	2.03 ± 1.04
	∑n-3 PUFAs (% of total fatty acids)	3.23 ± 1.72	7.96 ± 1.95	3.65 ± 1.84
**n-6/n-3 Index**		8.50 ± 2.83	4.14 ± 0.69	10.19 ± 4.88

Note. Data are presented as the mean ± SD. SFA: saturated fatty acids; MUFA: monounsaturated fatty acids; PUFA: polyunsaturated fatty acids; VLDL: Very Low-Density Lipoprotein.

**Table 6:** Comparison of the fatty acid composition between serum and erythrocyte membrane phospholipids.

	Fatty Acid	Red Blood Cells	Serum	*p*-Value (Wilcoxon Signed-Rank Test)	Serum-Erythrocyte Membrane Difference (95% CI)
	14:0 (Myristic acid)	0.49 ± 0.35	0.34 ± 0.96	0.009	−0.15 [−0.52, 0.22]
	16:0 (Palmitic acid)	21.20 ± 4.60	28.08 ± 2.60	<0.001	6.88 [5.12, 8.63]
	18:0 (Stearic acid)	11.99 ± 2.70	11.25 ± 4.72	0.761	−0.74 [−2.75, 1.28]
	22:0 (Docosanoic acid)	0.60 ± 0.39	0.03 ± 0.09	<0.001	−0.58 [−0.72, −0.43]
**MUFA**	14:1 (n-5)	0.24 ± 0.26	0.51 ± 0.37	0.008	0.26 [0.08, 0.45]
	16:1 (n-9)	1.20 ± 2.08	0.32 ± 0.81	0.041	−0.89 [−1.75, −0.03]
	18:1 (n-9) (Oleic acid)	16.96 ± 4.96	15.70 ± 2.49	0.001	−1.26 [−3.10, 0.58]
**PUFA-n6**	18:2 (n-6) (Linoleic acid)	15.47 ± 3.60	25.87 ± 3.29	<0.001	10.40 [8.97, 11.83]
	20:2 (n-6)	0.14 ± 0.19	0.16 ± 0.19	0.639	0.02 [−0.09, 0.13]
	20:3 (n-6) (DHGLA, dihomo-γ-linolenic acid)	1.57 ± 0.67	1.96 ± 0.82	0.048	0.40 [0.04, 0.76]
	20:4 (n-6) (AA, Arachidonic acid)	13.30 ± 3.15	9.04 ± 2.03	<0.001	−4.26 [−5.61, −2.92]
**PUFA-n3**	18:3 (n-3)	0.45 ± 0.68	0.02 ± 0.07	0.003	−0.44 [−0.69, −0.18]
	22:4 (n-3)	0.22 ± 0.50	0.22 ± 0.38	0.793	0.00 [−0.19, 0.20]
	22:5 (n-3) (DPA, docosapentaenoic acid)	1.03 ± 0.55	0.96 ± 0.83	0.821	−0.07 [−0.50, 0.35]
	22:6 (n-3) (DHA, docosahexaenoic acid)	3.13 ± 1.03	2.03 ± 1.04	0.001	−1.09 [−1.67, −0.52]

Note. Data are presented as the mean ± SD. Paired differences are expressed as serum-erythrocyte membrane values, with 95% confidence intervals. MUFA: monounsaturated fatty acids; PUFA: polyunsaturated fatty acids; CI: confidence interval.

**Table 7:** Correlation between the frequency of ultra-processed food consumption, physical activity levels, and circulating and membrane fatty acids.

		(UPF) r_s_	(Physical Act.) r_s_
**SFA**			
	VLDL	0.337 (*p* = 0.064)	0.010 (*p* = 0.957)
	Red blood cells	0.477 (*p* = 0.007)	−0.363 (*p* = 0.045)
	Serum	0.208 (*p* = 0.262)	0.180 (*p* = 0.333)
**MUFA**			
	VLDL	−0.184 (*p* = 0.322)	−0.183 (*p* = 0.324)
	Red blood cells	−0.343 (*p* = 0.059)	0.030 (*p* = 0.873)
	Serum	−0.399 (*p* = 0.026)	−0.193 (*p* = 0.298)
**PUFA-n6**			
	VLDL	0.475 (*p* = 0.007)	0.086 (*p* = 0.646)
	Red blood cells	−0.173 (*p* = 0.352)	0.250 (*p* = 0.175)
	Serum	0.311 (*p* = 0.089)	0.218 (*p* = 0.239)
**PUFA -n3**			
	VLDL	−0.503 (*p* = 0.004)	0.090 (*p* = 0.630)
	Red blood cells	−0.409 (*p* = 0.022)	−0.183 (*p* = 0.324)
	Serum	−0.298 (*p* = 0.103)	0.269 (*p* = 0.143)
**n-6/n-3 Index**			
	VLDL	−0.164 (*p* = 0.378)	0.082 (*p* = 0.661)
	Red blood cells	0.302 (*p* = 0.099)	0.071 (*p* = 0.704)
	Serum	−0.090 (*p* = 0.629)	0.253 (*p* = 0.170)

Note. SFA: saturated fatty acids; MUFA: monounsaturated fatty acids; PUFA: polyunsaturated fatty acids; VLDL: very-low-density lipoprotein; UPF: ultra-processed foods. Statistical measure used: Spearman’s rank correlation coefficient (rs). Exact *p*-values are reported in parentheses.

**Table 8:** Comparison of PUFA levels according to physical activity level.

		Low PAL	Moderate PAL	High PAL	H	*p*-Value	Sizeε^2^ (95% CI)
**PUFA-n6**							
	VLDL	26.75 [20.62–30.77]	30.55 [25.57–34.50]	26.60 [22.55–31.30]	1.042	0.594	0.000 [0.000, 0.269]
	Red blood cells	32.15 [30.15–35.05]	32.95 [30.60–35.88]	33.30 [32.65–35.50]	1.458	0.482	0.000 [0.000, 0.314]
	Serum	33.10 [31.45–38.27]	36.90 [36.00–38.65]	37.80 [36.45–39.30]	1.625	0.444	0.000 [0.000, 0.396]
**PUFA-n3**							
	VLDL	2.15 [1.60–3.08]	4.25 [3.20–4.92]	3.20 [2.35–4.20]	3.997	0.136	0.071 [0.000, 0.455]
	Red blood cells	9.05 [7.78–9.80]	8.00 [6.98–8.70]	8.00 [7.20–8.80]	1.569	0.456	0.000 [0.000, 0.338]
	Serum	1.85 [0.25–3.23]	4.30 [3.73–5.52]	3.80 [3.35–4.80]	4.679	0.096	0.096 [0.000, 0.444]
**n-6/n-3 Index**							
	VLDL	11.15 [9.69–12.31]	7.32 [5.86–7.59]	7.63 [6.74–9.88]	4.709	0.095	0.113 [0.000, 0.466]
	Red blood cells	3.65 [3.23–4.23]	4.07 [3.78–4.27]	4.09 [3.83–4.55]	1.618	0.445	0.000 [0.000, 0.397]
	Serum	13.70 [11.00–18.65]	8.16 [6.85–9.62]	9.70 [7.81–10.62]	2.870	0.238	0.036 [0.000, 0.459]

Note. Data are presented as the median [interquartile range]. PAL: physical activity level; PUFA: polyunsaturated fatty acids; VLDL: very-low-density lipoprotein; CI: confidence interval. Differences between physical activity groups were assessed using the Kruskal–Wallis test. Effect size was estimated using epsilon-squared (ε^2^) and is presented with 95% confidence intervals. The n-6/n-3 index was calculated only when ∑n-3 PUFA values were available and greater than zero.

## Data Availability

The data presented in this study is available on request from the corresponding author. The data is not publicly available due to maintaining the privacy of participants.
